# Understanding the impact of vitamin D supplement formulation, quality and provision to older adults in UK residential care homes†

**DOI:** 10.1039/d5pm00003c

**Published:** 2025-05-05

**Authors:** N. Rombel, C. Lim, A. Majeed, S. Abdul-Jabbar, G. R. McClelland, S. A. Jones

**Affiliations:** a Institute of Pharmaceutical Science, School of Cancer and Pharmaceutical Sciences, King's College London London UK Stuart.jones@kcl.ac.uk +44 (0)207 848 4506; b Centre for Pharmaceutical Medicine Research, Institute of Pharmaceutical Science, School of Cancer and Pharmaceutical Sciences, King's College London London UK

## Abstract

Supplying vitamin D supplements to all older adults is beneficial and cost-effective. However, operationalising this supply to residents in long-term care is problematic. This study aimed to understand the challenges of vitamin D supplement provision by auditing the extent of supplementation, measuring the quality of the supplements and investigating the attitudes towards supplement provision in UK care homes. This case study investigated the supply of vitamin D supplement formulations in four UK care homes and analysed the vitamin D content of nine formulation types. It employed semi-structured interviews with care home stakeholders to understand attitudes toward vitamin D supply. Across the nine analysed products, there was >50% variability in their quality (75–137% of the label), but 44% of supplements were of medicinal grade. One tablet from a food-grade product contained 167% vitamin D, and one medicinal-grade tablet only contained 70% vitamin D. Interviews with care home staff highlighted four challenges to providing supplements: the perceived responsibility of healthcare professionals to supplement, difficulties obtaining prescription medications, the absence of national/local strategies, and the financial burden. This study demonstrated sub-optimal vitamin D supplement supply to care home residents, with staff unclear about who was responsible for choosing the correct type of vitamin D supplement, who paid for it, and who was to supply it. This study suggests a new approach to delivering vitamin D supplements to older adults is needed.

## Abstract 2

The study demonstrates sub-optimal vitamin D supplement supply to care home residents (Created in BioRender. Jones, S. (2025) https://BioRender.com/sxhxl10).

## Graphical abstract

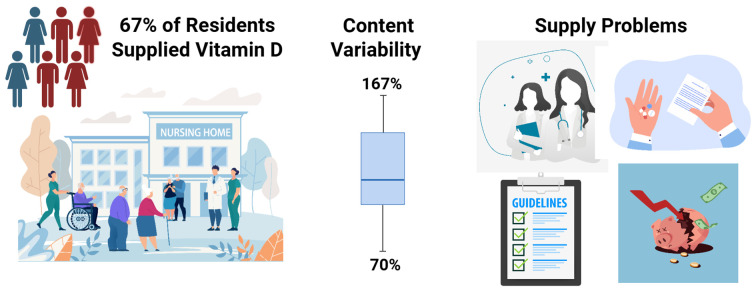

## 1. Introduction

Vitamin D has gained increasing public and academic interest, partly owing to its potential to alleviate the health impact of respiratory infections such as COVID-19.^1^ It is an essential nutrient in two primary forms: cholecalciferol (vitamin D_3_) and ergocalciferol (vitamin D_2_). It has a diverse range of biological actions, from modulating immune responses to controlling cell cycles, but its primary function is the regulation of calcium and phosphate metabolism. Vitamin D_3_ is synthesised endogenously in the skin following exposure to ultraviolet B radiation in sunlight (wavelength 280–315 nm) and can also be acquired from dietary sources, although to a much lesser extent.^2^ An individual's vitamin D status can be assessed by measuring the circulating concentration of its metabolite, 25-hydroxyvitamin D (25-OHD), with a value below 25 nmol L^−1^ indicating deficiency.^3,4^

The risk of vitamin D deficiency is especially pronounced in older adults as the ability to synthesise vitamin D in the skin decreases with age, and limited mobility further restricts exposure to the sun.^5^ For those living in long-term care facilities, lifestyle factors such as dietary intake and less time spent outdoors exacerbate the risk of deficiency.^6^ Results from the National Diet and Nutrition survey found that 10–20% of older adults have poor vitamin D status, which increases to 38% of people living in institutionalised care.^7^

Although recent large prospective cohort studies that investigated the effects of oral vitamin D on cancer, diabetes and cardiovascular disease (VITAL study, 25 000 adults over 5 years, the ViDA study, 5000 adults over 3.3 years, and the D2d study, 2423 participants over 2.3 years) have failed to show a statistically significant effect of vitamin D supplementation on disease outcomes, there is strong evidence that vitamin D supplementation has health benefits and is cost-effective in older adults.^8^ For example, a 2018 meta-analysis of five RCTs yielded a significant reduction in total cancer mortality by 13% (95% confidence interval, CI: 4–21%) and using this data, recent research in Germany has shown that medical-grade supplementation for older adults has the potential of reducing almost 30 000 deaths per year.^9^ This body of evidence has resulted in many national health authorities recommending vitamin D supplementation for large sections of the population; for example, the UK government recommends 10 μg (400 IU) for all adults, including populations deemed at risk, *i.e.*, the frail and institutionalised.^3,10^ However, even when governments recommend specific supplements, operationalising the provision of these supplements can be problematic. For example, work in Danish nursing homes showed that although knowledge of government recommendations was high (88% of questionnaire respondents), adherence to supplement provision was poor (<40% of respondents).^11^

One contributing factor to the difficulties in implementing vitamin D supplementation in elderly populations in institutional care is the dual regulatory classification of vitamins as both a nutritional supplement and a medicine.^12,13^ This complicates the supply and can result in “shifting” the burden between stakeholders. National medicine regulatory agencies regulate medicines, but national food standard authorities regulate foods, and these two bodies often have different requirements for supply and quality. The difference in the quality of supplements may mean that the clinical impact of the supplements may also differ.^14^ In addition, vitamin D can be fortified in food and these three ways to provide vitamin D can be confusing for care homes as they aim to maintain their residents’ health through providing healthy food and good medical care. It is not clear which of these two areas vitamin D fits.^15^ Previous work has suggested that care providers “medicalise” vitamin D provision, which hinders supplementation due to the requirement for a prescription.^16^ In addition, it has been shown that clinicians can be hesitant to provide a prescription for medical-grade supplements as food-grade supplements are available.^17^ However, previous work has not considered the quality of vitamin D supplements and this needs to be integrated into understanding the challenges in vitamin D supply so that safe and effective solutions can be proposed.

This study aimed to better understand the challenges of vitamin D supplement provision by auditing the extent of supplementation, the attitudes towards provision, and the quality of the supplements provided to residents in UK care homes. A mixed methods case study approach was employed, using qualitative interviews with care home staff members to understand attitudes towards supplement provision, a quantitative audit to understand the extent of provision, and laboratory analysis of the supplements to understand the quality of the preparations supplied.

## 2. Methods

### 2.1 Product quality assessment: vitamin D extraction and qualification

Vitamin D_3_ was extracted from the products by either pouring the liquid from the container directly, using the unit dose apparatus supplied with the product, releasing the liquid from soft gelatine capsule preparations by removing the exterior casing, or crushing the tablets. For the liquids, 1 mL of an internal standard vitamin D_2_ (50 μg mL^−1^) was added, and the contents were quantified by the RP-HPLC method using a calibration curve (see ESI† for the methods). For the solids, again, the internal standard was added, but this time with hexane (100 mL), water (50 mL), and methanol (50 mL) to perform a liquid–liquid extraction. The hexane layer was removed on a rotary evaporator (Büchi Rotavapor 114, Switzerland) at 40 °C. The final residue was dissolved in methanol and quantified by RP-HPLC using peak height (peak height was used due to the close elution times of vitamin D_2_ and D_3_). The percentage of vitamin D_3_ content compared to the label claim of all the supplements identified in the care home audit was performed. The results were compared to the ranges outlined for medicinal products in the British Pharmacopoeia (BP) and European Commission.^4,5^ Statistical data comparison was performed using one-way ANOVA, Turkey's HSD, after checking the data was normally distributed using Levene's test, in SPSS (IBM, USA). Statistically significant differences were annotated with asterisks to represent *p < 0.05 and **p < 0.01.

### 2.2 Case study methodology

This was an exploratory case study to understand better the current practice of vitamin D supplementation in elderly residential care homes in Southeast England. It employed semi-structured interviews with stakeholders of four non-profit residential care home organisations. Participants were identified using purposeful and convenience sampling from a pool of potential candidates suggested by the homes’ senior management teams. Inclusion criteria for the interviewees were those directly involved in the medicines or nutritional management of older adult residents. A quantitative audit of vitamin D products from the four care homes and subsequent laboratory investigation of the product quality from those care homes followed the interviews. Ethical approval for the interviews was gained from the KIng's College London Ethics Committee (MRSU-21/22-26885).

### 2.3 Case study data collection and analysis

Once written consent had been obtained, eligible individuals for the interviews were contacted *via* email and invited to participate in the case study. The interviewees were a care home manager, two deputy managers, a chef, and a support service manager drawn from various care homes. Interviews were conducted *via* the Zoom video conferencing platform (Zoom Video Communications, Inc.) between December 6th and 31st, 2021 (during the COVID-19 pandemic). The 30 min interviews were video recorded and transcribed verbatim utilising Otter.ai transcription software (AlSense, Inc.). NR analysed the text through immersive reading, identifying key themes, and then coding the text using central themes and sub-themes. SAJ verified codes and themes through an iterative process during a series of meetings to reduce bias. To understand the types of vitamin D preparations currently in use, a qualitative data collection tool (Table S1†) was created and sent to staff members before the interview process to record the supplements used during the week of the interview from the 4 care homes.

## 3. Results

### 3.1 Vitamin D_3_ preparations used in care homes and their quality

**Fig. 1:**
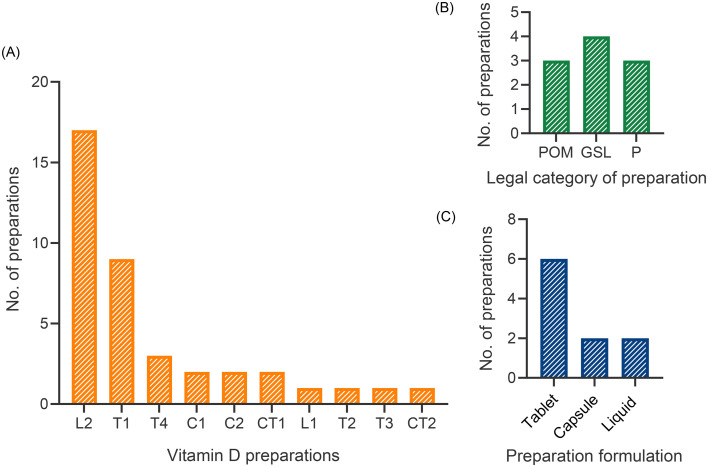
(A) Number of vitamin D preparations supplied within two residential care homes by type of vitamin D preparation. Preparations has been coded to maintain brand impartiality (B) Number of vitamin D preparations by legal category of preparation. (C) Number of vitamin D preparations by type of preparation formulation. L = liquid, C = capsule, T = tablet, CT = chewable tablet, G = granules. Total number of preparations *n* = 41. POM is prescription only medicine, GSL is general sales list item, and P is pharmacy only item.

Data collected on the types of vitamin D preparations used in the residential homes revealed that 41 of the 61 residents audited were using a vitamin D product (67%). Ten different preparations were used: two liquid formulations, two soft gelatine capsules, and six tablets (Fig. 1). Vitamin D drops were the most frequently supplied preparation, used by 17 residents, followed by chewable tablets, used by nine residents. It is important to note that the vitamin D drops most frequently found in the care homes were the products provided by the NHS in their initiative to supplement vitamin D to “clinically extremely vulnerable” populations during COVID-19. There was a range of different types of products, including three prescription-only medicines (POM) three pharmacy-only medicines (P) and four general sales list (GSL) items (Fig. 1). All preparations supplied within the care homes were initially provided through a prescription. Tablet formulations, including chewable tablets, were the most common formulation type, with six preparations in use by the care home residents (Fig. 1).

**Fig. 2:**
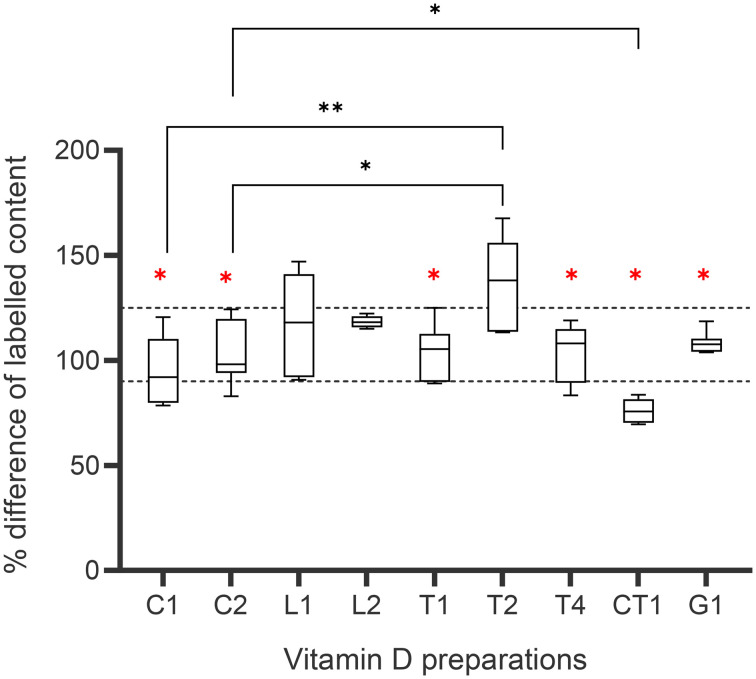
Vitamin D_3_ recovery from the different products used in the care homes. Red * represents licensed medicinal products, black * denotes statistical difference (*p < 0.05, **p < 0.01), dotted lines delineate British Pharmacopoeia (BP) acceptance criterion for medicinal products (90–125%). Data represents the mean ± one standard deviation.

The RP-HPLC method was validated according to the ICH guidelines (Fig. S1†). Only nine of the ten supplements used in the care homes could be purchased. The POM and P medicines were obtained from a wholesale distributor, and the GSL products from a pharmacy. They were all in date; thus, they were assumed to have been stored correctly and of good quality prior to analysis (the 10^th^ was no longer available). Three batches of all the products were subjected to RP-HPLC analysis to ascertain the content of vitamin D_3_*vs.* the label claim (%) (Fig. 2 and Table S1†). Six of the nine analysed products were licensed medicinal-grade products, five had a content within the BP specified limit, and one product was outside this range (90% to 125%). The vitamin D content of the non-medicinal grade products ranged from 75–137%. The average content of the GSL products remained within the food grade limits of 80–150%. One of the food-grade tablets gave a content of 167%, which raises quality concerns. The chromatograms of the analysed cholecalciferol products (Fig. S2–S6†) displayed typical elution of the vitamin D_3_ analyte at 16 min and vitamin D_2_ at 16.25 min. The chromatographic peaks were characteristically sharp for both the vitamin D_3_ and internal standard.^18^ The internal standard's mean percentage recovery (%) for all preparations was 98.8% ± 8.39.

### 3.2 Interviews

Five different employees of four care homes were interviewed. Table 1 outlines participant demographics, including roles and care home type. The qualitative analysis uncovered two major themes and eight sub-themes (Tables 2 and 3).

**Table 1:** Interviewee demographics

Coding	Role	Establishment type
CM	Chef manager	Residential, dementia, respite care
SSM	Support services manager	Residential, dementia, day care
CHM	Care home manager	Residential, dementia, respite care
DM1, DM2	Deputy managers	Residential, dementia, respite care

**Table 2:** Representative quotations

**Sub-theme 1. Understanding of vitamin D as medically important for care home residents**
(1) “*…we can see how vitamin D has had an impact on residents in a good way because it has improved how they are, in terms of even being more energetic and doing more walking, especially after COVID when people lost a lot of abilities*” (CHM)
(2) “*If we have things which then can help them to sustain their well-being, then we should be…holding on to it so that people can have a better life at the end of their life*” (CHM)
(3) “*I say that vitamin D is something which should be considered for everyone in care homes and should continue. The government should really look into that because people's well-being is improved*” (CHM)
**Sub-theme 2. Awareness of national vitamin D guidance**
(4) “*Yes, we are aware of that guidance that came out. It's certainly something that's been talked about in the care industry for quite a while now…but certainly with COVID and with more research done into it, I think that did kind of push it back*” (SSM)
(5) “*…especially with the pandemic, there was a lot of information flying about. After COVID there were so many initiatives…so you have to sort of grab whatever you can to try to enhance the care of the people you look after*” (CHM)
**Sub-theme 3. Blanket supplementation resulting in challenges to implementation**
(6) “*Had that been confirmed that it was required? Do we make assumptions that most elderly people in care would be deficient in it? So there was a lot of talk in the care industry around the assumptions you would have to make*” (SSM)
(7) “*I can't just put someone on something without a reason, so I understand the balance of trying to protect people but also the balance of how those logistics also delay and affect good things from being done quicker*” (CHM)
**Sub-theme 4. Generational boundaries and personal choice incite resistance to vitamin D supplementation**
(8) “*… there's still this reluctance to take supplements or tablets. If you've got a condition that you're prescribed a medication for I think people kind of accept that. We're still talking about a generation that wouldn't take tablets for tablets taking sake. That can sometimes be a barrier*” (SSM)
(9) “*The generation we are looking after some of them are really anti-medication, so they will not take something if they don't feel that they need it. Sometimes it's just very difficult, especially if they've got capacity, to try to convince them that it is in their best interest…*” (CHM)
(10) “*That was one of the issues when we were looking at trialling this liquid supplement with the prescribing dietitian. Firstly, it was getting consent*” (SSM)

**Table 3:** Representative quotations

**Challenge 1. Financial burden falls on care home residents; a limiting factor to supplement supply**
(1) “*At the end of the day, we wouldn't be able to fund it as an organisation. It would have to be the residents that pay for it themselves*” (SSM)
(2) “*What about those who don't have the finances? So, then we also go on about equal opportunities…and then you find out that some people might not have it because they don't have the money to buy it*” (CHM)
(3) “*I've talked to the prescribing dietitian for [the county] about a vitamin D supplement that was a liquid…but again, it all came back to we couldn't progress it because of the cost implications*” (SSM)
**Challenge 2. Requesting prescriptions from general practitioners poses problems**
(4) “*I think it's about the logistics…if I go to my doctor and say, ‘I've got 36 people to be reviewed to see whether they can be on vitamin D′, that can take weeks before I get answers. So that's why I'm saying that the processes can take longer, whereas, if it is what is being decided, then shouldn't we just put it on prescription?*” (CHM)
(5) “*…if you're doing that for one person then it's doable. When you've got to sit and speak to a doctor about 80 residents, they don't have time for that. Then they've got to do 80 prescriptions*” (DM1)
(6) “*Now every time you speak to a GP they'll say, ‘Oh yeah, they can be on this one, but there's this cheaper version’… they're always looking for the cheaper version, but does the cheaper version have the same results as the one which costs a bit more?*” (CHM)
**Challenge 3. No national or local strategic plan for provision of vitamin D supplementation**
(7) “*The government gave a free supply to all care home residents for four months, so they recognise the importance of it, but they give it for four months and then stop it. That gives us the wrong message. If it's that important at the time, why does it not remain important?*” (SSM)
(8) “*I don't know, is it manpower? Is it money? You don't know what it is and why is ends up like that because the initiative comes, it's enforced…and then after a couple of months, three months, it just stops*” (CHM)
(9) “*It seemed more like a spur of the moment thing. It was like ‘we don't know what to do during COVID, let's chuck something at the residents to make it look like we're doing something’*” (DM1)
(10) “*If an initiative is good then, before people are withdrawn from it, there needs to be a plan of how it's going to carry forward*” (CHM)
(11) “*…if it runs out that can't be the end of it, especially when we are talking about possibly another wave of COVID coming in which means people might be indoors or in isolation for longer periods and not able to access the community*” (CHM)
(12) “*It's just been a crazy couple years and it just feels like the care sector has been left hung out to dry*” (DM1)
**Challenge 4. Perceived responsibility of the government and healthcare professionals**
(13) “*It shouldn't be us keeping on trying to convince everyone to help us keep people on it. Medical people should be coming on board and saying that people need it, saying ‘we have reviewed this person and they should be on it because they're in a care home’…*” (CHM)
(14) “*It should be initially started by the government really. I mean, we'd have to keep it up if we were told ‘this is what we need to do’… at [our organisation] we'd follow the rules or anything that we had been told*” (DM1)
(15) “*But surely it should have been a government push thing*” (DM1)

**Theme 1: The decision to take vitamin D supplements within residential care homes is complex, personal, and driven by consent.**

Vitamin D was widely understood as medically important to the residents and critical for achieving positive health outcomes within this vulnerable population; for example, one participant noted, “*we should be… holding onto it so that people can have a better life at the end of their life*” (Sub-theme 1, Quotation 2). Most care home stakeholders expressed an awareness of the UK government's guidance for vitamin D supplementation, with 4 of the 5 participants having heard of it previously (Table 2). Despite this, the provision of vitamin D was complex, and numerous factors contributed to the decision to supply it. The interviewees were unanimous in their opinion that there was confusion around the supply and the decision-making process that proceeded it (Sub-theme 3). Importantly, the decision to take supplements such as vitamin D was seen as personal. There was the perception that central government guidance to initiate mandatory cholecalciferol supplementation in care homes was at odds with the active personal decision process within the homes (Sub-theme 3). It was noted by one interviewee that you cannot simply “*put someone on something without a reason*” and it was thought that blanket use of supplementation would be tethered with a range of “*assumptions you would have to make*” (Sub-theme 3, Quotations 3 and 4) to provide vitamin D within care homes. Supplementation was often linked to roles in the care homes, for example, the chef manager considers food to be their domain and vitamin supplementation is the domain of the healthcare professionals. Surprisingly, the care home staff suggested many care home residents were not familiar with the concept of taking supplements nor the need for them, which was referred to as an “anti-supplement mentality”. Some linked this to an “anti-medication” mentality, which was common in the home and fuelled resistance to supplement usage amongst the elderly population (Sub-theme 4).

**Theme 2: Challanges to the provision of vitamin D supplements by residential care homes.**

The challenges of providing vitamin D, in any form, to elderly residents within the care homes were discussed in all the interviews (Table 3). The cost implications of funding supplements and medicines seemed to unpin a cautious approach. Links were made between the provision of supplements and the costs to residents. These links were suggested to limit their use; for example, one interviewee said, “S*ome people might not have it because they don't have the money to buy it*” (Challange 1, Quotation 2). Requesting free supplements *via* prescription provided by the National Health Service, although appearing to solve the cost issues, posed other difficulties, including logistical challenges and the perception that it was the opinion of general practitioners that cheaper, over-the-counter medications should be used instead. One of the interviewees touched on the issue that over-the-counter medications may not have the same efficacy as those on prescription, saying, “*…but does the cheaper version have the same results as the one which costs a bit more?*” (Challange 2, Quotation 6). This brought together the elements of supply, costs and quality, which were woven into all the interviews in different forms.

Although interviewees understood the importance of providing supplements, they did not know how to achieve this practically. The discontinuation of the NHS initiative, which provided a 4-months’ supply of free vitamin D supplements to care homes in England during COVID-19, coupled with the continued government guidance to ensure vitamin D status is maintained, led to a feeling of concern of who was picking up the supply bill, “*it just feels like the care sector has been left hung out to dry*” (Challange 3, Quotation 13). Several interviewees expressed the need for a new approach to providing vitamin D moving forward, with a strong consensus that this should be the government's responsibility through healthcare professionals. Care homes would “*keep it up if… told, ‘this is what we need to do’*” (Challange 4, Quotation 15), emphasising that current vitamin D guidance was not enough to change current practices.

## 4. Discussion

According to a population survey in England, one in three men and women living within institutionalised care are deficient with serum 25-OHD concentrations below 25 nmol L^−1^.^19^ Over the last 20 years, a paucity of literature has considered this important topic. The problems of deficiency in this special population have been echoed by the Scientific Advisory Committee on Nutrition (SACN), which found that 37–38% of institutionalised adults were deficient.^3^ In this study, only 67% of older adults in the care homes audited were supplied with vitamin D supplements despite the recent free provision in the UK during COVID-19. This falls short of supplying all residents and highlights a significant problem with vitamin D supply.

All of the interviewees in the current case study spoke to the perceived professional boundaries that exist; the supply of vitamins is considered the responsibility of healthcare workers rather than the care home staff. Fears of overstepping their role had consequences on providing vitamin D, and the result was no care-home-wide supplementation as per the UK government guidance.^19^ The medical framing of vitamin D imposes limitations to its supply, which are deep-seated and not easy to remove.^16,20^ According to the UK Health and Social Care Act (2008), dietary supplements can only be administered to residents “when supplied by a healthcare professional”.^15^ However, general practitioners are advised against routine prescription of vitamin preparations in primary care by NHS clinical commissioning groups.^16^ The consequence is that vitamin D is not reaching those populations in most need – the older age adults who are institutionalised. The literature suggests that the ‘medicalisation’ of vitamins and foods is a well-recognised phenomenon in medical practice,^21,22^ and evidence from this study suggests that it also exists in the social care sector.

The interviews highlighted that personal choice and autonomous decision-making are linked to excellent care. This generates a need to provide the recipient with the correct vitamin D supplement, but care home staff thought this was outside the scope of their role.^20^ The desire of care providers to integrate elements of choice into vitamin D provision explains, at least in part, why the existence of “blanket” guidance, which does not appear to be personalised to the needs of care home residents, does not result in a complete change in practice within care homes.^23^

Responses to the interview questions suggested that cholecalciferol supplements, or supplements in general, were unimportant in the residents’ daily lives. The care home staff cited that an anti-medication mindset was prevalent. Previous work reported that limited vitamin D knowledge^24,25^ and the unpalatability of vitamin and calcium combination tablets^20^ can be barriers to supplement use. However, the study population did not include care home residents, and this work's applicability to those in long-term care is questionable. Surprisingly, one study found that despite only 43.5% of participants taking vitamin D supplements, 2/3^rd^ of the population were inclined to consume fortified foods.^25^ The positive attitudes to food fortification could support the potential implementation of fortification strategies in care homes, presenting an alternative for this population in which an “anti-supplement” mentality exists. Although the national guidance for catering in residential care broadly states the need for vitamin D supplementation, it does not address how this may be implemented in practice and thus requires clarification.^26^ Many countries have implemented mandatory food fortification programmes with great success, including Canada, USA,^27^ Finland,^28^ and Germany.^24^ National policies in Finland advise manufacturers to include 10 μg and 0.5 μg vitamin D per product in fat spreads and milk products, respectively, and these have proven to be an effective solution for reducing vitamin D deficiency at the population level with a mean 25(OH)D concentration rise from 47.6 to 65.4 nmol l^−1^ in adults over a 10-year period.^28^ However, because the provision of fortifying foods is difficult to achieve practically, this can widen discrepancies in intake that are difficult to account for when highly deficient residents present to clinicians.

When oral supplements are used, the various sources of the supplements can lead to the use of products of varying quality. Two interviewees identified the potential difference in the quality of pharmaceutical-grade *vs.* food-grade supplements as an important factor to consider in the supply of vitamin D within care homes, and this has not previously been reported. The staff clearly understood that the different means of providing supplements were likely to result in different outcomes, but there was no clear understanding of how quality impacted the outcome. This was unsurprising given the dearth of literature in this area. The laboratory analysis of the products found in the care homes indicated that medicinal products were of significantly higher quality. Vitamin D product quality is particularly important as the absorption of this vitamin is highly variable. Previous work has suggested that a single dose of oral vitamin D can vary in its absorption by up to 20-fold.^29^ This variability, in combination with significant variations in the product label claim, could result in product safety issues with high concentrations of vitamin D, leading to hypercalcemia or sub-therapeutic doses that do not achieve the intended pharmacological effect.^29^ Label claim ranges of 41–165% in the UK,^30^ 8–177% in the Netherlands,^31^ 66–145% in Canada,^32^ and 9–146% in the United States^33^ are in line with the data from the current work. More data is required to establish the clinical consequence of poor vitamin D supplement quality on the vitamin D status of older adults in care homes. However, if the differences in the quality of non-medicinal supplements are found to be clinically important, new approaches to providing vitamin D supplements to older adults should be considered.^34,35^

## 5. Strengths and limitations of the study

This study provides insights into the supply of vitamin D supplements in 4 residential care homes managed by Quantum Care, in one area of England, and it should be generalised with caution. However, at the time of the study, there were no institutional guidelines for the supply of vitamin D in the care homes (after the study, this changed); therefore, the single supplier of care was not thought to introduce significant bias. The generalisability is also impacted by the relatively low number of interviews conducted. However, the study's findings accorded with previously published data, and they were consistent across the different care homes, suggesting that the highlighted themes were applicable to other institutionalised care settings. Using a mixed methods approach was a strength of the study, and the alignment of the findings from the interviews, the audit of vitamin D supplements and the quantitative assessment of the supplement quality was unique to this work and made its findings impactful.

## 6. Conclusion

This study suggests that current UK government guidelines do not result in effective vitamin D supplementation for older adults. Care homes were unclear about who was responsible for choosing the correct type of supplement, who paid for it, and who was to supply it. Due to these challenges, a range of vitamin D supplements were provided to care home residents, resulting in variable quality. The challenges outlined in this work could be solved by supplying vitamin D supplements *via* a single route, *e.g.*, food fortification or prescription-only products, but the choice of this supply route is problematic because, as exemplified by the recent UK report on vitamin D food fortification, the impact of the supplementation method, on vitamin D status is unclear.^36^ Further work should investigate the clinical impact of different forms of supplementation in care for older adults, and alternative vitamin D formulations should be designed to facilitate a high-quality supply to vulnerable populations such as older adults.

## Author contributions

C. L.: data curation, writing – reviewing and editing, N. R.: investigation, data curation, writing – reviewing and editing. A. M.: investigation, data curation, S. A. J.: reviewing and editing. G. M.: reviewing and editing. S. J.: conceptualisation, methodology, data curation, original draft preparation, supervision, reviewing and editing.

## Declaration

Ethical approval and guidelines for the work was gained from the KCL ethics committee (MRSU-21/22-26885). Informed consent was obtained from interviewees.

## Conflicts of interest

S. J has received funding from Patch MD and Vitamax Wholesalers and holds a patent on vitamin D phosphate (WO2022/084669). NR, CL, AM, SAJ and GM have no competing interests.

## Supplementary Material

PM-002-D5PM00003C-s001

## Data Availability

The data supporting this article have been included as part of the ESI.†
