# Supplementary material for: Understanding the impact of vitamin D supplement formulation, quality and provision to older adults in UK residential care homes
Source: RSC Pharm. 2025 May 5;2(4):683–90. doi: 10.1039/d5pm00003c (PMC12090889; doi:10.1039/d5pm00003c)
Supplement: PM-002-D5PM00003C-s001 [file PM-002-D5PM00003C-s001.pdf]

Supplementary Information (SI) for RSC Pharmaceutics.
This journal is © The Royal Society of Chemistry 2025

Supporting Information

Product quality assessment: materials

HPLC-grade water, methanol (MeOH), acetonitrile (ACN), trifluoroacetic acid (TFA), cholecalciferol (vitamin D_3) and ergocalciferol (vitamin D_2) were obtained from Sigma-Aldrich (Steinheim, Germany). HPLC-grade hexane (C6H14) was purchased from ThermoFisher Scientific (Waltham, Massachusetts). Vitamin D_3 products were commercially available in the UK and included Colecalciferol Capsules (Alissa Healthcare, England), Vitamin D Drops (Provitavit, England), Ddrops (iHerb, USA), and Vitamin D_3 (Valupak, UK). Ultra-pure water was supplied by PURELAB Ultra purification system (ELGA LabWater, UK).

Product quality assessment: method validation

Reverse phase high-performance liquid chromatography (RP-HPLC) analysis was carried out using a high-performance liquid chromatograph Series 1100 equipped with an auto-sample injector, a pump, and a diode array detector (Agilent Technologies, USA). Chromatographic separation of the analytes was performed on a Phenomenes Kinetex Biphenyl column (250 x 4.6 mm, 5 Ȧ diameter) with the collection and analysis of output peaks using a ChemStation data acquisition system (Agilent Technologies, USA). Following sonication for 20 minutes (GT Sonic Ultrasonic Cleaner, China), a gradient eluent method of mobile phase A (water: methanol, 80:20 and 0.1% TFA) and mobile phase B (ACN: methanol: water, 70:20:10 and 0.1% TFA) was conducted. The composition of mobile phase B used to achieve peak separation was as follows: 50% (0 min), increased to 90% (10 -15 min) followed by subsequent decrease to 50% (17 - 20 min), for a total run time of 20 min. A flow rate of 1.0 mL/min and injection volume of 50 µL were employed with UV detection carried out at 265 nm.

The RP-HPLC method was validated in accordance with the principles for analytical procedures defined by the ICH Harmonized Tripartite Guideline Q2(R1) using the verification criteria of linearity, accuracy, precision, limit of quantification, and limit of detection. Linearity was assessed through the construction of calibration curves with five calibration standards across a range of concentrations (3.03 - 296 µg/mL). The linearity (R^2) of the calibration graph was calculated by least-square regression analysis. Three quality control samples of known vitamin D3 concentration were analysed in triplicate to assess method accuracy. The suitability of the chromatographic system was assessed using peak symmetry and theoretical plate number.

Product Quality Supplementary Results

Both peak area and peak height were found to be effective methods to quantify vitamin D_3 using HPLC analysis. Vitamin D_3 was eluted at a retention time of 16.08 min and the internal vitamin D_2 at 16.29 min. As the two peaks were not fully resolved, peak height was employed to determine the vitamin D_3 content of the test supplements.

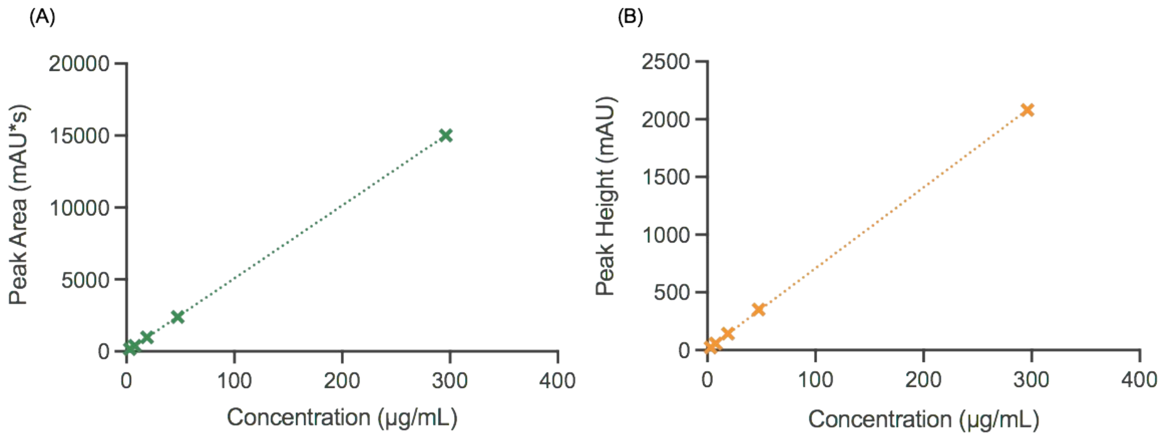

Figure S1. RP-HPLC calibration curves for vitamin D_3 with (A) peak area (mAU*s) and (B) peak height (mAU) plotted against concentration (µg/mL). Equation of the line of regression: (A) $y = 50.693x + 1.5058$ ($R^2 = 0.9999$) and (B) $y = 7.0077x + 7.9699$ ($R^2 = 0.9999$). Values represent the mean ± standard deviation of n = 15 samples detected at 265 nm.

The method was verified as "fit for purpose" according to the ICH guidelines (*Table 2*). Based on the five calibration standards, the calibration graphs yielded the following regression equations: (A) $y = 50.693x + 1.5058$ and (B) $y = 7.0077x + 7.9699$. Calibration linearity at 0.9999 was acceptable and method accuracy was established at 104.95 ± 3.7 and 105.35 ± 6.74 for peak area and peak height, respectively. The repeatability showed a percentage CV of below 2%, i.e. the ICH limit. The LOD and LOQ values for peak height of 0.57 µg/mL and 1.73 µg/mL, respectively, demonstrated acceptable sensitivity of the chromatographic method.

Table S1. Vitamin D product quality (* indicates medicinal product)

Supplement	Theoretical vitamin D_3 conc. (µg/ml)	Measured vitamin D_3 conc. (µg/ml) (n = 6 ± SD)	Proportion of label claim (%)	BP limit recommendation
**Soft Gelatin Capsule Formulations**				
**(C1) Cholecalciferol Capsule (20 µg)*	20	19.00 ± 3.30	95.02 ± 16.48	90 – 125 %
**(C2) Cholecalciferol Capsule (25 µg)*	25	20.66 ± 3.05	103.29 ± 15.27	90 – 125 %
**Liquid Formulations**				
(L1) Liquid Vitamin D_3 (25 µg)	25	29.40 ± 6.35	117.59 ± 25.38	90 – 110 %
(L2) Vitamin D drops (5 µg)	10	11.84 ± 0.27	118.42 ± 2.70	90 – 110 %
**Solid formulations**				
(T3) Vitamin D_3 (25 µg)	25	34.30 ± 5.36	137.20 ± 21.45	90 – 125 %
**(T1) chewable tablets (10 µg)*	10	10.44 ± 1.26	104.42 ± 12.60	90 – 125 %
**(T4) film coated tablets (10 µg)*	10	10.28 ± 1.37	102.81 ± 13.68	90 – 125 %
**(CT1) chewable tablets (22 µg)*	22	16.69 ± 1.25	75.86 ± 5.70	90 – 125 %
**(G1) effervescent granules (11 µg)*	11	11.89 ± 0.52	108.07 ± 4.77	90 – 125 %

Table S2. Quantitative data collection tool for the audit of vitamin D_3 preparations used in care homes.

	**Name of Vitamin D Supplement (i.e. Colecalciferol tablets, SunVit-D3, Aciferol D3)**	**Type of Vitamin D Supplement (i.e. Tablet, Capsule, Solution)**	**Strength of Vitamin D Supplement (IU/µg) (i.e. 400 IU, 10 µg)**	**Total number of residents supplied the supplement (No.)**	**How often is the supplement taken? (i.e. once a day, once a week)**
**1.**					
**2….**					

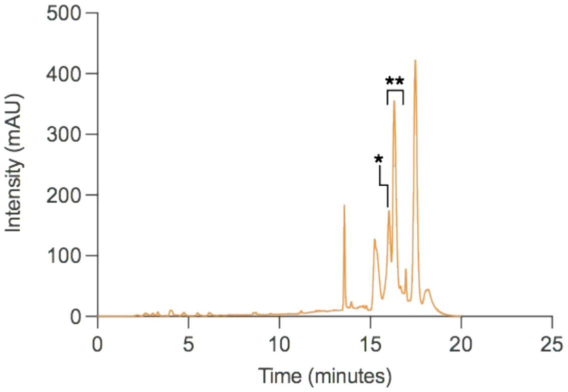

Figure S2. RP-HPLC chromatogram of C1 sample and vitamin D_2 internal standard on a Phenomenes Kinetex Biphenyl column (250 x 4.6 mm, 5 Å diameter) using mobile phase A: water/methanol (80:20, 0.1% TFA) and mobile phase B: ACN/methanol/water (70:20:10, 0.1% TFA) in a gradient eluent method. Volume of injection set to 50 µL, detection at 265 nm. * denotes vitamin D_3 sample, ** denotes vitamin D_2 internal standard.

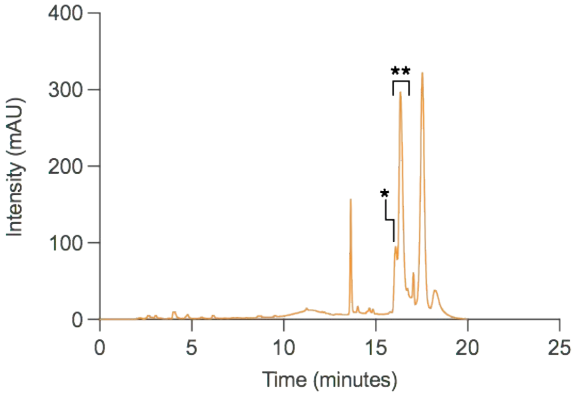

Figure S3. RP-HPLC chromatogram of C2 Capsule sample and vitamin D_2 internal standard on a Phenomenes Kinetex Biphenyl column (250 x 4.6 mm, 5 Å diameter) using mobile phase A: water/methanol (80:20, 0.1% TFA) and mobile phase B: ACN/methanol/water (70:20:10, 0.1% TFA) in a gradient eluent method. Volume of injection set to 50 µL, detection at 265 nm. * denotes vitamin D_3 sample, ** denotes vitamin D_2 internal standard.

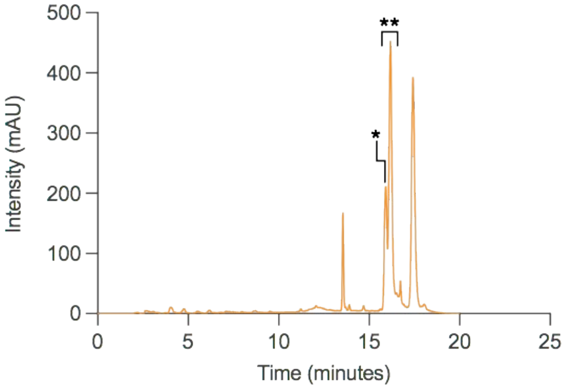

Figure S4. RP-HPLC chromatogram of L1 Vitamin D3 sample and vitamin D_2 internal standard on a Phenomenes Kinetex Biphenyl column (250 x 4.6 mm, 5 Å diameter) using mobile phase A: water/methanol (80:20, 0.1% TFA) and mobile phase B: ACN/methanol/water (70:20:10, 0.1% TFA) in a gradient eluent method. Volume of injection set to 50 µL, detection at 265 nm. * denotes vitamin D_3 sample, ** denotes vitamin D_2 internal standard.

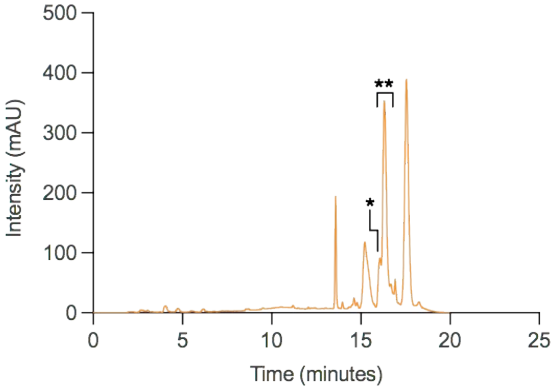

Figure S5. RP-HPLC chromatogram of L2 Vitamin D Drops sample and vitamin D_2 internal standard on a Phenomenes Kinetex Biphenyl column (250 x 4.6 mm, 5 Å diameter) using mobile phase A: water/methanol (80:20, 0.1% TFA) and mobile phase B: ACN/methanol/water (70:20:10, 0.1% TFA) in a gradient eluent method. Volume of injection set to 50 µL, detection at 265 nm. * denotes vitamin D_3 sample, ** denotes vitamin D_2 internal standard.

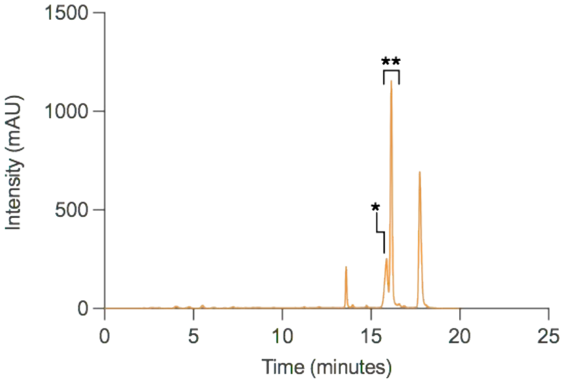

Figure S6. RP-HPLC chromatogram of T3 sample and vitamin D_2 internal standard on a Phenomenes Kinetex Biphenyl column (250 x 4.6 mm, 5 Å diameter) using mobile phase A: water/methanol (80:20, 0.1% TFA) and mobile phase B: ACN/methanol/water (70:20:10, 0.1% TFA) in a gradient eluent method. Volume of injection set to 50 µL, detection at 265 nm. * denotes vitamin D_3 sample, ** denotes vitamin D_2 internal standard.
